# Suspected Buruli ulcer cases in Tonkolili District, Sierra Leone– a prospective cohort study

**DOI:** 10.1007/s15010-025-02548-2

**Published:** 2025-05-21

**Authors:** Jonathan Vas Nunes, Lars Wassill, Giulia Mönnink, Abdul-Mac Falama, Hanna Mathéron, Amara Conteh, Maxwell Sesay, Aminata Sesay, Håkon Bolkan, Martin P. Grobusch, Frieder Schaumburg

**Affiliations:** 1 Masanga Medical Research Unit (MMRU), Masanga, Sierra Leone; 2 Center of Tropical Medicine and Travel Medicine, Department of Infectious Diseases, Amsterdam University Medical Centers, Location AMC, Amsterdam Public Health, Amsterdam Infection & Immunity, Amsterdam, The Netherlands; 3 Albert Schweitzer Hospital, Dordrecht, The Netherlands; 4 AmplexDiagnostics GmbH, Gars-Bahnhof, Germany; 5 Ministry of Health and Sanitation, Freetown, Sierra Leone; 6 Spaarne Gasthuis Hospital, Heemstede, The Netherlands; 7 Department of Public Health and Nursing, Norwegian University of Science and Technology (NTNU), Trondheim, Norway; 8 Clinic of Surgery, St. Olavs Hospital HF, Trondheim University Hospital, Trondheim, Norway; 9 CapaCare, Trondheim, Norway; 10 CapaCare, Heemstede, The Netherlands; 11 CapaCare, Masanga, Sierra Leone; 12 Institute of Medical Microbiology, University Hospital Münster, Münster, Germany; 13 Resident General Surgery, Medical Doctor in Global Health & Tropical Medicine, Department of Surgery, Amsterdam University Medical Center, Location AMC. Meibergdreef 9, Amsterdam, 1105 AZ The Netherlands

**Keywords:** Buruli ulcer, *Mycobacterium ulcerans*, Sub-Saharan Africa, Neglected tropical diseases, Diagnosis, LAMP

## Abstract

**Purpose:**

There is a high burden of chronic ulcers in Sierra Leone. However, (early) diagnosis and treatment are challenging. Data on endemicity of *Mycobacterium ulcerans* is limited to WHO reports from 2008 to 2011.

**Methods:**

Patients presenting with wounds at Masanga Teaching Hospital were included in a prospective cohort study and scored following the WHO clinical list for Buruli ulcer (BU). Wounds were screened for *M. ulcerans* by selective culture on solid and liquid media and loop-mediated isothermal amplification (LAMP) of the *M. ulcerans* specific *IS2404*.

**Results:**

Between July 2019 and November 2020, 159 patients were included. The median age was 41 years (range: 2–92), 34% (54/159) were female and 56% (89/159) were literate. The median duration of a wound before admission was 12 months (range: 0-720 months), 87% (137/159) of lesions were below the knee. Wounds of 37% (58/159) of the patients were clinically scored as ‘(very) likely to be Buruli ulcer’. Seven out of 72 patients tested by LAMP were positive for *IS2404*, two showed specific melting curves. None of the wound swabs yielded a positive culture for *M. ulcerans*. Ninety-eight (62%) patients had a wound-related surgery during this study, 101 (63%) of patients were improving or healed at the time of discharge.

**Conclusions:**

The prevalence of BU based on the WHO scoring system is high in Sierra Leone. National and international awareness, training of healthcare workers, development of in-country bacteriology as well as the furthering of robust molecular and immunological assays could reduce the burden of this neglected tropical disease.

## Introduction

Severe and chronic wounds are common in Sierra Leone and associated with mortality, morbidity, lack of income and catastrophic expenditures [1–3]. The health care system in Sierra Leone is fragile and lacks diagnostic and therapeutic means. Patients often receive suboptimal treatment for their wound, further aggravating their condition.

Buruli ulcer (BU), an often debilitating but neglected tropical skin disease caused by *Mycobacterium ulcerans*, is reported in mostly tropical countries across the globe, especially in West-Africa (e.g. Ghana, Cameroon, Benin, Burkina Faso, Liberia, Guinea) [4–10]. Data on the endemicity of BU in Sierra Leone is limited: the WHO reports only one case of BU in 2008 and 28 in 2011 [11–13]. Numbers of unreported cases are most likely high. A worldwide downward trend in the number of BU cases is reported by the WHO, with only 2121 notified cases in 2022 versus 3213 in 2012 (66%) [14]. Some West-African countries such as Benin and Ghana report a similar decline. Lalo district in Benin reported only one case of BU among 10,000 inhabitants in 2017 versus 22 cases per 10,000 in 2006 [15–17]. Although no data from Sierra Leone or neighbouring countries Liberia and Guinea support a similar decline, a study in 2021 on 21 severe wound patients in Sierra Leone was unable to confirm any *M. ulcerans* infection [18, 19].

Mycolactone, a toxin of *M. ulcerans*, causes necrosis, often leading to ulceration or deeper tissue invasion such as osteomyelitis or late complications like contractures and squamous cell carcinomas [20–22]. Early antimicrobial treatment of BU may prevent ulcer progression, deformities or worse– conditions often necessitating surgical treatment or amputation. Diagnosis is a challenge, especially in low resource settings such as in Sierra Leone [23]. Several tools are recommended by the WHO to enable (early) diagnosis, such as a clinical scoring system, staining for acid-fast bacilli, culturing, histopathology, and nucleic acid amplification techniques like PCR or loop-mediated isothermal amplification [LAMP]) [24]. However, these modalities have several challenges. A clinical scoring system such as from the WHO or one of an expert panel as described by Eddyani in 2018, can reach a sensitivity of up to 92%, exceeding any current laboratory test [25, 26]. However, these scoring systems are highly dependent on clinical experience with BU and regional endemicity. Staining for acid-fast bacilli and histopathology have a sensitivity of up to 59% and culturing up to 51%, whilst PCR of previously untreated BU can reach up to 90% sensitivity [27]. Promising innovations in fluorescent thin layer chromatography and point-of-care or rapid diagnostic testing to detect for instance mycolactone, are investigated [28, 29]. Most of these tools, however, are not readily available in low resource settings. In addition, knowledge on BU is scarce and most hospitals lack availability of medication and human resources for surgical treatment.

The objectives of the study were to determine the prevalence of suspected BU in patients at a wound clinic in Tonkolili District, Sierra Leone and to identify interventions to improve BU related wound care in Sierra Leone. In addition, this study aimed at evaluating the use of a ready-to-use LAMP-assay targeting *IS 2404*, a diagnostic tool that is particularly suitable in resource limited settings [30].

## Methods

### Study setting

Masanga Teaching Hospital (MTH), a former Leprosy hospital in rural Tonkolili District, Sierra Leone, serves as a referral centre for wound care. Up to 300 wound patients are seen annually for wound treatment. All patients are seen both by a physician and a dedicated wound dresser. At the time of this study, clinical diagnosis was supported by limited diagnostic tools, including basic haematology and biochemistry tests as well as analogue X-rays and serology rapid tests for HIV, syphilis and hepatitis, and basic microscopy including gram-staining and Ziehl-Neelsen staining for acid fast bacilli.

### Study population

Patients with open wounds were included in this prospective cohort study if they were admitted or seen as out-patient at Masanga Hospital between 10 July 2019 and 30 November 2020. Exclusion criteria were not consenting, or presentation with a surgical site infection within 30 days after surgery. Risk factors for BU, e.g. pain, persistence of the wound, manual labour in stagnant water and smoking, diabetes (as assessed by fasting blood glucose level with an Accu-Chek handheld device) and clinical symptoms were recorded in a standard case report form. Photos of the wound on primary presentation as well as on monthly intervals and/or discharge were taken.

### Microbiology

Samples were taken at inclusion by thoroughly swabbing the central part of the wound and, if possible, the tissue beneath the edges of the wound after removal of topical treatment, bandages, and necrosis. Sterile cotton tips in Amies medium were used for culture and Polyurethane-foam sponge tips in liquid Amies medium for LAMP (Transswab, MWE, Corsham, UK). After sampling, all swabs were stored in the Masanga Hospital laboratory at 2–7 °C. LAMP for *IS 2404* and bacteriological culture for wound pathogens, including *M. ulcerans*, were not available in Sierra Leone at the time of the study. Therefore, all swabs were transported for culture and LAMP, roughly every three months, to the Institute of Medical Microbiology of the University Hospital in Münster, Germany. Transportation of swabs was done at room temperature; analysis was then performed directly upon arrival in Germany.

Culture for *M. ulcerans* was done following the WHO guideline for the “laboratory diagnosis of Buruli ulcer” [31]. Briefly, swabs were decontaminated with the N-acetyl-L-cysteine-sodium hydroxide method. Resuspended sediments were dissolved in 1 ml distilled water. Suspension (0.2 ml) was cultured on Löwenstein-Jensen agar (BD BBL Löwenstein-Jensen medium, BD) at 30 °C for 6 months and checked for growth every 14 days. The rest of the suspension (0.8 ml) was cultured in liquid medium (Middlebrook, BD BBL MGIT, BD, Sparks, Maryland, USA) at 30 °C for 6 months and subcultured (1 ml) on Löwenstein-Jensen medium (BD) for another 6 months.

### Loop-mediated isothermal amplification (LAMP)

The WHO identified LAMP targeting the the multicopy insertion sequence (IS) 2404 as a promising tool for a point of care testing of *M. ulcerans* in settings with limited resources [30]. The LAMP-assay was developed using the primer sequences described elsewhere [32]. For DNA extraction, 25 µl of the liquid Amiens medium from a patient’s swab were added to 500 µl of „resuspension and lysis fluid“ (RALF buffer, AmplexDiagnostics, Gars-Bahnhof, Germany) and boiled for 10 min at 99 °C. The supernatant (25 µl, each) was transferred into two corresponding tubes (one with *IS2404*-specific primers, the other with an inhibition control to detect possible inhibition of the LAMP reaction due to inhibitory substances in the patient sample) of the test strip containing the lyophilized reagents. Tests were run on a Genie II device (Optigene Ltd, Horsham, UK) at 65° C for 25 min. Amplification was measured by real-time fluorescence detection using the intercalating dye contained in the lyophilized master mix of the assays. Test results were automatically calculated, using an algorithm based on fluorescence signal and steepness of amplification curve, and reported by the integrated software on the Genie II instrument. At the end of the test run, a melting curve analysis of the resulting amplification products was carried out to check the specificity of the amplification reaction. Amplicons of the *IS-2404* assay show a peak of the melting curve at 91.5 °C while the peak of the melting curve of the inhibition control amplicon is at 82.0 °C.

### Statistical analyses

Statistical analyses were performed with Excel v16.81 and Stata v16. Wounds were scored according to the WHO ‘Skin NTDs clinical and treatment form’, with one modification: all patients scored a one for evolution, as only the duration of the wound was questioned [26]. Patients were grouped by their total clinical score, according to the WHO cut-off values for BU: ‘very unlikely to be BU’ (10–13 points), ‘unlikely BU’ (14–16 points), ‘likely BU’ (17–20) and ‘very likely BU’ (21–24). An association between BU clinical score and risk factors (e.g. usage of alcohol, smoking, frequent exposure to stagnant water, HIV, diabetes) was assessed by logistic regression. The significance level was set at 0.05.

## Results

### Population

A total of 159 patient were included. None were excluded. The median age was 41 years (2–92), 34% was female, 56% was literate, 37% smoked and 28% drank alcohol regularly (Table 1). 8% had HIV, 24% had diabetes. Patients presented with wounds lasting between two days and 60 years (median 12 months), four patients (2,5%) presented within one week. 87% of lesions were located below the knee. 46% of patients were regularly exposed to stagnant water. Nineteen patients (12%) had more than one lesion. Prior to presentation at the wound clinic, two (1%) patients underwent an amputation, and seven (4%) patients had a surgical debridement performed. Fourteen (9%) patients reported a skin graft in their medical history; four of them had a modified WHO clinical score of 17 or above (Table 1).

**Table 1:** Patient characteristics

		All (*n* = 159)	WHO clinical score for BU		OR (95%CI)	*p*-value
			(very) likely BU 17–24 (*n* = 58)	(very) unlikely BU 10–16 (*n* = 101)		
Demographics	Median age in years (range)	41 (2–92)	35 (2–78)	50 (2–92)		
	<15	10 (6%)	8 (14%)	2 (2%)	#	#
	15–49	89 (56%)	45 (78%)	46 (46%)		
	50-above	60 (38%)	7 (12%)	53 (52%)		
	Sex (female)	54 (34%)	15 (26%)	39 (39%)	1.8 (0.9–3.7)	0.104
	BMI (median)	21	22	21	1.0 (0.9-1.0)	0.880
Highest education	Illiterate	70 (44%)	19 (33%)	52 (51%)	0.5 (0.2–0.9)	0.033 *
	Primary	20 (13%)	19 (33%)	2 (2%)		
	Secondary	51 (32%)	13 (22%)	38 (38%)		
	College/University	18 (11%)	10 (17%)	8 (8%)		
Medical history	Alcohol (yes)	44 (28%)	16 (28%)	29 (29%)	1.0 (0.5–2.2)	0.929 *
	Smoking (yes)	59 (37%)	18 (31%)	42 (42%)	0.7 (0.3–1.4)	0.322 *
	Median packyears (range)	1.50 (0.13–6.88)	1.50 (0.13–6.88)	2.02 (0.08–3.75)		
	HIV positive	12 (8%)	5 (9%)	7 (7%)	1.2 (0.4–4.1)	0.698
	Median glucose level in mmol/L (range)	6.2 (1.2–24.0)	6.0 (2.2–14.5)	6.2 (1.2–24.0)	1.0 (0.5–2.2)	0.957
	Diabetes (> 6.9 mmol/L)^b^	38 (24%)	14 (24%)	24 (24%)	1.0 (0.5–2.2)	0.957
	History of wound surgery (yes) ^a^	22 (14%)	5 (9%)	17 (17%)		
	Fever (> 37,5 C)	6 (4%)	3 (1%)	3 (3%)	#	#
	Treatment by a traditional healer (yes)	89 (56%)	30 (52%)	61 (60%)	0.6 (0.3–1.2)	0.140
Number of lesions	1	140 (88%)	54 (93%)	86 (85%)	#	#
	> 1	19 (12%)	4 (7%)	15 (15%)		
Characterization of lesions	Undermined edge ^c^ (yes)	3 (2%)	3 (5%)	0	#	#
	Pain (more than a little)	8 (3%)	4 (7%	4 (4%)	#	#
	Lymph node enlargement (yes)	12 (8%)	0	12 (12%)	#	#
Duration of lesion ^c^	Median duration of lesion in months (range)	12 (0-720)	3 (0–60)	17 (1-720)		
	<3 months	35 (22%)	28 (48%)	7 (7%)	#	#
	3–6 months	23 (14%	11 (19%)	12 (12%)		
	>6 months	101 (64%)	19 (33%)	82 (81%)		
Location of lesion ^c^	Ankle and foot	66 (42%)	14 (24%)	52 (51%)	#	#
	Between ankle and knee	71 (45%)	24 (41%)	47 (47%)		
	Above knee	22 (14%)	20 (34%)	2 (2%)		
Exposure to stagnant water	Never	86 (55%)	35 (60%)	52 (51%)	0.7 (0.4–1.4)	0.385
	1–2 days a week	64 (40%)	24 (41%)	41 (41%)		
	3–7 days a week	9 (6%)	1 (2%)	8 (8%)		

* p-value calculated for patients > 14 years

^#^ Not calculated as already part of the clinical scoring system

^a^ wound surgery prior to this presentation, including Split Skin Grafting (SSG), debridement, amputation, surgery for osteomyelitis

^b^ WHO mean fasting blood glucose cutoff values [33]

^c^ largest lesion

Eighty-five of the patients had a modified WHO clinical score for BU of 17 or above, indicating (very) likely BU (Table 1). Swabbing and LAMP testing was performed irrespective of differential diagnoses. Subject to local availability of swabs, only patients arriving from 5 August 2020 until 30 November 2020 were routinely tested for *M. ulcerans* by LAMP (*n* = 72) of which 10% (7/72) had a positive LAMP according to the integrated algorithm. Two (3%) of them showed a typical peak of the melting curve at 91.5 °C suggesting a specific amplification of *IS2404* and therefore the presence of *M. ulcerans*. These two patients had a WHO BU score of 16 and 20. Swabs for *M. ulcerans* culture were taken from all patients (*n* = 159) but no sample was culture-positive. Three patients were tested for acid-fast bacilli with Ziehl-Neelsen staining, one was positive and diagnosed with leprosy.

94% of all patients and 87% of patients with a WHO clinical score for BU of 17–24 were above 15 years old. 33% (19/58) of patients with a BU score above 16 were illiterate, compared with 44% amongst the whole study population (OR = 0.5, 95% CI: 0.2–0.9; *p* = 0.033). No significant association were found between the BU clinical score and sex, BMI, usage of alcohol, smoking, frequent exposure to stagnant water, topical traditional treatment, HIV status and diabetes (Table 1).

### Wound management and clinical course of infection

51% of patients (*n* = 82) reported to have used at least one type of systemic antibiotics prior to visiting the hospital. Use of topical antibiotics prior to visiting the hospital was reported by 38% of patients (*n* = 61). One person was recently treated with rifampicin-isoniazid-pyrazinamide-ethambutol (RHZE) prior to initial presentation (BU clinical score 15). One person was treated with RHZE against tuberculosis during admission to the wound clinic (clinical score 19,), one received rifampicin with clindamycin during wound treatment (clinical score 17). Two people reported to have had RHZE during follow up after wound treatment (clinical scores of 15 and 16). No one had received clarithromycin.

Ninety-eight (62%) of patients had any form of wound-related surgery whilst being enrolled in this study. Most performed procedures were split skin grafts (*n* = 48) and debridements (*n* = 46). A total of 101 (63%) patients were improving or healed at the time of discharge. Of the 58 people with a clinical score for BU of 17 or more, 19 (33%) had at least one surgical debridement, nine (16%) were amputated, 17 (29%) received a skin graft and eight (14%) had osteomyelitis related surgery. Thirty-three (57%) were healed, eight of them after amputation, and 13 (22%) were improving. Four (7%) were palliative or died due to complications of their wound and three (5%) discharged themselves due to lack of money, no improvements or fear of amputation (Table 2).

**Table 2:** Wound management and clinical outcomes

		All (*n* = 159)	WHO Clinical Score 17–24 (*n* = 58)	WHO Clinical Score 10–16 (*n* = 101)
WHO treatment category ^a^	I	16 (10%)	5 (9%)	11 (11%)
	II	77 (48%)	25 (43%)	52 (51%)
	III	66 (42%)	28 (48%)	38 (38%)
Surgical treatment	Any form of wound related surgery	98 (62%)	37 (64%)	61 (60%)
Surgical procedures	Surgical debridement	46 (29%)	19 (33%)	27 (27%)
	Amputation	28 (18%)	9 (16%)	19 (19%)
	Split Skin Graft	48 (30%)	17 (29%)	31 (31%)
	Osteomyelitis surgery	12 (8%)	8 (14%)	4 (4%)
Outcome at discharge	Healed	57 (36%)	25 (43%)	32 (32%)
	Healed (but amputated)	18 (11%)	8 (14%)	10 (10%)
	Improving	26 (16%)	13 (13%)	13 (13%)
	Palliative	9 (6%)	2 (3%)	7 (7%)
	Deceased (likely due to wound)	9 (6%)	2 (3%)	7 (7%)
	Deceased (likely not due to wound)	1 (1%)	0	1 (1%)
	No money to continue treatment	7 (4%)	1 (2%)	6 (6%)
	Left for lack of improvement	2 (1%)	1 (2%)	1 (1%)
	Unknown	1 (1%)	0	1 (1%)
	Left for fear of amputation	4 (3%)	1 (2%)	3 (3%)
	Other	18 (18%)	5 (9%)	13 (13%)

^a^ Reference [34]

**Fig. 1:**
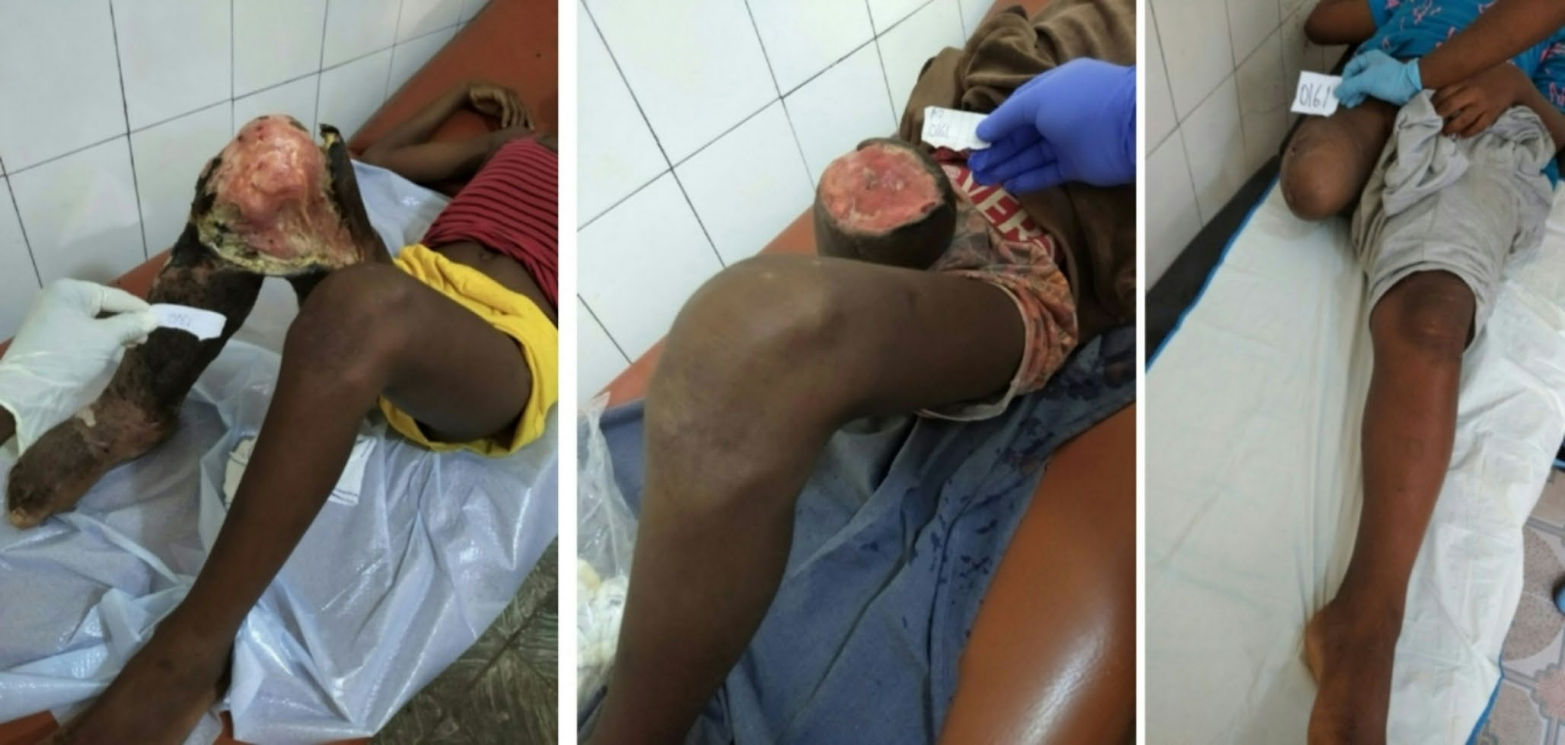
Case vignette box of two patients with suspected Buruli ulcer. Case 1 (WHO clinical score 20– ‘likely to be BU’): a 14-year old male, literate, non-smoker, with no apparent medical history. The wound on his right knee started with a boil, four months prior to presentation. He was treated by a traditional healer for one months with leaves on the wound and self-bought diclofenac, ampicillin/cloxacillin and albendazole. He was afebrile. His right leg was oedematous, with an offensive smell and the ulcer had undermined edges. The wound (diameter: 25 cm) was painful (four on a scale of one to five) and there was a contracture in his knee. He was tested negative for HIV. A differential diagnosis of BU was made, including common bacterial or fungal skin infections, yaws, cutaneous leishmaniasis, pyoderma gangrenosum, pyomyositis, chronic arthritis, osteomyelitis or malignancyMeta. He was amputated three days after admission because the joint had become dysfunctional. He was discharged with a closed stump. He tested positive for *Mycobacterium ulcerans* by LAMP for *IS 2404* with a specific peak of the melting curve at 91.5 °C

An example of the course of disease is highlighted as case vignette for one patient with a clinical score of 20, who was tested LAMP positive for BU (Fig. 1– Case vignette box).

## Discussion

We performed a prospective cohort study on patients with chronic wounds and found a high proportion of (very) likely BU patients (36%) based on the WHO scoring system.

### Reflections on local and regional epidemiology

Although children 15 years and below account for 48% of BU cases in Africa, in this study only 14% of patients with a clinical score likely to be BU were below 15 years old [35]. A possible explanation is that only 10/159 patients (6.3%) in this study were < 15 years old, even though 39% of people in Sierra Leone are 0–14 years old [36]. One reason could be that adults in Sierra Leone work more likely than children in the fields, often in stagnant water, and undergo minor trauma to the lower extremities. Another explanation could be that children are less likely to seek care, and there are more children in Sierra Leone undiagnosed as they have had a spontaneous regression or (successful) traditional treatment or are currently living with a chronic ulcer or osteomyelitis.

### Reflections on diagnostic challenges– old and present

The differential diagnosis of a (chronic) ulcer in Sierra Leone is challenging, and does not only include BU but also, for example, common bacterial skin infections caused by e.g. *Staphylococci* or *Streptococci*, (deep) fungal infections, leprosy, yaws, cutaneous leishmaniasis, tropical ulcers, malignancies and arterial or venous pathology. An additional challenge to differentiate these wounds is a late presentation; only 22% of people with a wound in this study presented within 3 months and only 2.5% of people presented within one week. This late presentation complicates wound diagnosis, e.g. due to absence of characteristic nodules or undermined edges or smell, an increased likelihood of bacterial superinfection, antimicrobial resistance due to (out of hospital) antibiotics usage and spontaneous clearance of the original wound pathogen (such as BU). In addition, this delay may lead to polymicrobial wound pathogens, a common finding in chronic wounds in Sierra Leone and Ghana [3, 37, 38]. Even for BU patients that have been treated with streptomycin/rifampicin, a polymicrobial superinfection with a high bacterial load post-treatment is common [39].

For BU diagnosis in Sierra Leone, challenges in diagnosis are highlighted by a previous study in 2019, where none of 21 patients with a severe wound were found to be positive for BU when tested with dry swabs for PCR, ethanol swabs for thin layer chromatography, culture swabs and two biopsies each for a direct smear examination [18]. Point of care test can improve the diagnosis of BU and the detection of *M. ulcerans*. We therefore applied a LAMP targeting the *IS2404*, as suggested by the WHO [30]. Two of the samples were positive (i.e. specific melting curve peak at 91.5 °C). Another five yielded positive amplification curves but were recognized as non-specific by the melting curve analysis. Depending on the extraction methods and sets of primers, the LAMP can reach a sensitivity and specificity of 91.5% and 97.6%, respectively [40]. However, our examples illustrate the need for rigorous validation of methods, even if recommended methods and primers are applied.

Long standing ulcers, common in Sierra Leone due to a high unmet need for surgical (wound) care, may show spontaneous healing, false-negative results, sampling error and nucleic acid amplification inhibition [1, 41]. Another factor at play may be a relatively high number of patients presenting late with a secondary bacterial infection, in 51% of cases after systemic antibiotics prior to presentation and in 56% of cases after visiting a traditional healer. This may have led to unique microbiomes and an atypical clinical presentation, with e.g. more pain (atypical for BU) or lack of a characteristic BU smell (a study by Ayelo et al. found this smell to be strongly associated with a positive PCR result for BU) [37, 42, 43].

Combining clinical judgement of an expert panel with PCR confirmation has shown a sensitivity of up to 92% in Benin [25]. No additional variables were identified in this study to significantly predict a higher clinical BU score, although literacy rate was significantly higher amongst patients with a clinical score likely to be BU. This was still significant when analysing only patients above 14 years old. This may however reflect the low proportion of patients under 15 years old in our study.

### Clinical outcomes and traditional medicine

Traditional medicines are common in Sierra Leone. Whilst 56% percent of the patients admitted to having seen a traditional healer prior to visiting the wound clinic, perhaps more did so without wanting to admit this to the wound clinic staff. Frequently reported treatments are leaves or a paste on the wound, hot rub, or an animal skin on the wound. This last decade, interesting studies in e.g. Cameroon and Benin were conducted on the potential curative attributes of indigenous plants on BU [44–48]. In contrast to these potential curative attributes, however, a common perception amongst healthcare workers in Sierra Leone is that traditional wound care is potentially harmful by causing delay in presentation or by causing a secondary infection of an existing wound. Even if people would have been cleared of *M. ulcerans* (spontaneous, by indigenous plants or e.g. streptomycin/rifampicin), post-treatment polymicrobial superinfection is still a risk factor for late complications [39]. In addition, several reports exist on *M. ulcerans* in the environment in West-Africa [49–52]. One could therefore hypothesise that some BUs may be caused by traditional wound treatment with topical use of material contaminated with *M. ulcerans*. More research may be helpful to select those types of plants or topical (heat) treatments that are effective in the treatment of BU and those instances where ‘iatrogenic’ infection with *M. ulcerans* takes place.

### Improvement of wound care with local means and knowledge gaps

Wound care for BU in Sierra Leone may improve with further training of designated wound dressing teams and regional collaboration. In addition, Masanga Teaching Hospital has established a bacteriology laboratory and, in conjunction with the Masanga Medical Research Unit and several partners including the Ministry of Health and Sanitation (MoHS) of Sierra Leone, conducts further research on BU diagnosis in Sierra Leone.

### (Inter)national awareness & future ideas

BU is a challenging and often neglected tropical disease. This is especially true in the limited resource setting of Sierra Leone. Even at Masanga Hospital, a national referral centre for wound care, where dedicated wound dressers with training on BU see approximately 75–90 patients a day in the wound clinic, diagnostic tools are limited to staining and the WHO clinical scoring list [53]. Although there is some surgical expertise for wound debridement, skin grafting and amputation, rifampicin and clarithromycin as first line medical treatment are not always readily available.

Training of healthcare staff, including wound dressers, community health workers and officers and medical doctors in BU may be beneficial for (early) detection of BU in Sierra Leone. Especially when combined with more availability of bacteriology and molecular testing for BU within Sierra Leone. Installation of an expert panel for Sierra Leone, or participation in a regional expert panel may be pursued. In addition to this, research and funding partnerships may be created and (inter-)national awareness on the endemicity of BU raised. These actions all fit well in the Sierra Leone NTD Master Plan 2023–2027 of the Ministry of Health and Sanitation to reduce the number of BU patients in Sierra Leone [54].

### Limitations of this study

Firstly, due to unavailability of swabs, only 72 of 159 patients were tested by LAMP. Secondly, since the study is a single-centre study, findings might not be representative for Sierra Leone. Thirdly, although we tried to adhere as much as possible to the WHO diagnostic guidelines for BU, some deviations from the proposed WHO method (e.g. use of Amies medium instead of Dubos broth base, decontamination after shipment to Germany instead of right after sampling) might explain the non-detection of *M. ulcerans* by culture. Fourthly, as only the duration of the wound was recorded, no data was gathered on evolution of the wound. Therefore, all patients were given a score of 1 out of maximum 2 on this point of the WHO clinical scoring list. This may have underestimated the group likely or very likely to have BU.

## Conclusion

Our study suggests that BU is still prevalent in Sierra Leone. Fifty-eight out of a total 159 patients scored a ‘likely to be BU’ on the WHO clinical scoring list. Two out of 72 patients were tested positive for *M. ulcerans* by LAMP. National and international awareness, training of healthcare workers, development of in-country bacteriology and nucleic-acid amplification capacity and potential availability of (rapid) tests could reduce the burden of this neglected tropical disease.

## Data Availability

Data is provided within the manuscript. Additional data from the original database may be made available by the authors, upon reasonable request.
